# The correlation between endometrial thickness and outcome of in vitro fertilization and embryo transfer (IVF-ET) outcome

**DOI:** 10.1186/1477-7827-6-37

**Published:** 2008-09-02

**Authors:** Ahlam Al-Ghamdi, Serdar Coskun, Saad Al-Hassan, Rafat Al-Rejjal, Khalid Awartani

**Affiliations:** 1 Reproductive Medicine, Department of Obstetrics and Gynecology, King Faisal Specialist Hospital and Research Centre Riyadh, Kingdom of Saudi Arabia

## Abstract

**Background:**

To evaluate the relationship between endometrial thickness on day of human chorionic gonadotrophin administration (hCG) and pregnancy outcome in a large number of consecutive in vitro fertilization and embryo transfer (IVF-ET) cycles.

**Methods:**

A retrospective cohort study including all patients who had IVF-ET from January 2003–December 2005 conducted at a tertiary center.

**Results:**

A total of 2464 cycles were analysed. Pregnancy rate (PR) was 35.8%. PR increased linearly (r = 0.864) from 29.4% among patients with a lining of less than or equal to 6 mm, to 44.4% among patients with a lining of greater than or equal to 17 mm. ROC showed that endometrial thickness is not a good predictor of PR, so a definite cut-off value could not be established (AUC = 0.55).

**Conclusion:**

There is a positive linear relationship between the endometrial thickness measured on the day of hCG injection and PR, and is independent of other variables. Hence aiming for a thicker endometrium should be considered.

## Background

Assisted reproductive technology (ART) has been commonly used in infertility treatment over the last two decades. The high cost and relatively low implantation and pregnancy rates (PRs) in in-vitro fertilization (IVF) and intracytoplasmic sperm injection (ICSI) treatment cycles has led to a need to evaluate the predictors of success in these patients. One of the important factors is the endometrial receptivity. Endometrial thickness has been utilized as an indirect indicator for endometrial receptivity and is measured in the midsaggital plane during transvaginal ultrasound, which is considered as both atraumatic and simple [1]. The effect of endometrial thickness on pregnancy rates in ART patients has been evaluated by many authors [2-11], with controversial results. Some authors demonstrated a higher pregnancy rate at certain endometrial thickness [3,4,10-12], while others did not show a significant correlation between endometrial thickness and PRs in IVF/ICSI patients [5,7,8]. Other authors reported a threshold of <7 mm and/or >14 mm with a significant reduction in implantation rate and PR [2,6].

With these controversies, no conclusive cut-off value of endometrial thickness has been established in order to help clinicians in counseling the couple about the outcome. The reason for such controversy could be probably due to a relatively low number of cycles for patients with both extremes of endometrial thicknessess.

The aim of this study is to determine if there is any effect of endometrial thickness measured on the day of administration of human Chorionic Gonadotrphin (hCG) on pregnancy rate while analyzing large number of cycles, and if so, to identify a cut off value at which pregnancy rate is too low, hence helping clinicians in counceling the couples.

## Methods

All fresh cycles of IVF or ICSI conducted at King Faisal Specialist Hospital and Research Center IVF unit from January 2003 to December 2005 were identified from our electronic database and the charts were reviewed. The study was approved by the Ethics Committee of our hospital. All fresh IVF or ICSI treatment cycles that reached oocyte pick up and embryo transfer within the study period were included, women with known intrauterine anomalies were excluded from the study. Endometrial thickness was not used as a criteria for cancellation. Endometrial thickness was defined as the maximal distance between the echogenic interfaces of the myometrium and the endometrium and was measured in the midsagittal plane by two dimensional transvaginal ultrasound on the day of hCG administration.

Two protocols for pituitary down regulation were used, long or short protocol as previously described [1]. The medication for stimulation used in all cases was human menopausal gonadotrophin (hMG, Menegon^®^, Ferring, Germany). When at least three follicles were ≥ 18 mm, hCG 10,000 units was administered. The endometrial thickness was measured by the same sonographer and documented in the chart. Oocyte retrieval was performed 36 hours later. Fertilization was achieved by IVF or ICSI according to the indication. Cleavage stage embryos were transferred on day 3. Maximum two embryos were transferred under transabdominal ultrasound guidance with a full bladder. The patients were started on IM progesterone injections (Gestone, Nordic Pharma, UK) on the same day of embryo transfer for luteal phase support and continued till pregnancy test on day 15. Clinical pregnancy was confirmed by ultrasound observation of fetal cardiac activity two weeks after positive hCG test.

The patients were divided into two groups; those who got pregnant (group A) and those who did not (group B). Both groups were compared for the various parameters including age, body mass index (BMI), diagnosis, number of oocyte retrieved, length of stimulation, dose of hMG, fertilization rate, number of cleaved embryos, number of transferred embryos.

### Statistical analysis

Data were analyzed using SPSS version 14 software (Chicago, Ilin, USA). All tests were two tailed, and p < 0.05 was considered statistically significant. Continuous variables are presented as mean and SD and were tested by student's t-test. Comparisons of proportions were made by the chi-squared test. The effect of endometrial thickness on the pregnancy outcome was studied using multivariate analysis, where all other factors affecting the pregnancy outcome were controlled for. To determine the correlation between endometrial thickness, patient characteristics and treatment characteristics a stepwise logistic regression analysis was performed including (age of the patient, body mass index (BMI), endometrial thickness on day 3 of the cycle, duration of stimulation, dose of hMG needed, number of oocytes retrieved, number of cleaved embryos, and number of embryos transferred). The Receiver operating characteristic (ROC) analysis was used to evaluate an endometrial thickness that can predict pregnancy outcome.

## Results

**Figure 1:**
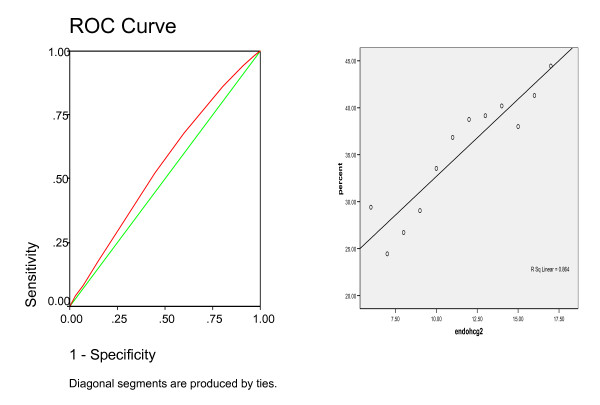
The ROC and linear regression curves.

A total of 2464 cycles were included in the study. Clinical pregnancy rate (PR) was 35.8%. 79% of the patients had undergone the long protocol. The pregnancy rate was 39.4% in the long protocol group vs 22.4% in the short protocol group. Compared to group B, group A patients were younger, required lower dose of hMG, had more medium sized and mature follicles, higher number of oocytes retrieved, higher number of oocytes fertilized, and higher number of cleaved embryos. Both groups had similar BMI, duration of stimulation, baseline endometrial thickness (measured on day 3 of the cycle before the start of hMG), and number of transferred embryos (Table 1). There was no statistical difference between the two groups in the primary infertility diagnosis (Table 2). Endometrial thickness measured on the day of hCG administration ranged between 5 – 20 mm, and was higher in cycles where pregnancy was achieved, with statistical significance (mean 11.6 vs. 11.3 mm, respectively, p < 0.0001). Pregnancy rate increased from 29.4% among patients with an endometrial thickness of ≤6 mm, to 44.4% among patients with an endometrial thickness of ≥17 mm (Table 3). (Figure 1) shows the positive linear correlation (r = 0.864) and ROC with an area under the curve (AUC) = 0.55. From this ROC a cut-off value of ≥11 mm would be suggested. When dividing the patients into two groups, group 1 with endometrial thickness of <11 mm, and group 2 with endometrial thickness ≥11 mm, PRs were 30.9% and 38.7% respectively, p = 0.001, RR = 1.25 (95%CI 1.12–1.41) (Table 4). Multiple logistic regression analysis indicated significant independent effects of age (P = 0.01), Type of protocol used (P = 0.0001), endometrial thickness on hCG day (P = 0.001), number of oocytes retrieved (P = 0.0001), number of cleaved embryos (P = 0.0001), and number of embryos transferred (P = 0.0001) on pregnancy rates.

**Table 1:** Demographic data

Characteristics	Group A mean ± SD	Group B mean ± SD	P value
Number of cycles (n)	882	1582	
Age (years)	30.27 ± 5.53	31.14 ± 5.38	0.0001
BMI (weight kg/height m^2^)	28.44 ± 4.58	28.32 ± 4.42	0.524
Long protocol # (%)	765 (39.4%)	1177 (60.6%)	< 0.0001
Short protocol # (%)	117 (22.4%)	405 (77.6%)	
Stimulation length (days)	10.92 ± 2.63	10.79 ± 2.46	0.228
Dose of hMG (ampoules)	37.67 ± 15.03	40.73 ± 16.54	< .0001
Endometrial thickness cycle day 3 (mm)	3.23 ± 1.22	3.21 ± 1.22	0.696
Endometrial thickness hCG day (mm)	11.64 ± 2.13	11.26 ± 2.17	< 0.0001
Number of medium sized follicles	8.08 ± 5.33	7.12 ± 5.31	< 0.0001
Number of mature follicles	7.92 ± 3.51	7.62 ± 3.69	0.0475
Number of oocytes retrieved	10.51 ± 5.43	9.86 ± 5.73	0.006
Number of fertilized oocytes	5.79 ± 3.23	4.97 ± 3.35	< 0.0001
Number of embryos	5.3 ± 2.82	4.44 ± 2.81	< 0.0001
Number of embryos transferred	1.88 ± 0.37	1.98 ± 0.26	0.001

**Table 2:** Diagnostic categories

Diagnosis	Group A (882)	Group B (1582)
Male factor 1763 (71.6%)	623 (70.6%)	1140 (72.0%)
Tubal factor 338 (13.7%)	114 (13.0%)	224 (14.2%)
Unexplained 213 (8.6%)	85 (9.6%)	128 (8.1%)
Others 150 (6.1%)	60 (6.8%)	90 (5.7%)

P = 0.319

**Table 3:** Pregnancy rates at different endometrial thicknesses

Endometrial thickness on day of HCG	Group A (n)	Group B (n)	Pregnancy rate
≤6 mm	5	12	29.40%
7 mm	11	34	24.40%
8 mm	35	96	26.70%
9 mm	70	171	29.00%
10 mm	162	321	33.50%
11 mm	140	240	36.80%
12 mm	174	275	38.80%
13 mm	130	202	39.20%
14 mm	82	122	40.20%
15 mm	38	62	38.00%
16 mm	19	27	41.30%
≥17 mm	16	20	44%
Total	882	1582	35.80%

**Table 4:** Pregnancy rates below and above 11 mm endometrial thickness

Endometrial thickness on day of HCG	Group A (n)	Group B (n)	Pregnancy rate
< 11 mm	283	634	30.90%
≥ 11 mm	599	948	38.70%
Total	882	1582	35.80%

P = 0.001

RR = 1.25, (95% CI 1.12–1.41)

## Discussion

This study is to our knowledge so far the largest in regards to sample size that addresses the effect of endometrial thickness on PR. The day of the stimulation cycle on which the endometrial thickness is measured to document adequate endometrial development has varied between authors. The most often used is the measurement taken on the day of hCG administration, but some authors have used the measurement that was taken on the day of oocyte retrieval or the day of embryo transfer in their studies, which makes it difficult to compare between studies. We have used the measurement taken on the day of hCG administration in our data. The change in endometrial thickness occurring during IVF stimulation has been evaluated by several authors [8,13,14]. Grant et al 2007, demonstrated a trend toward significance in the overall change in endometrial thickness between the baseline and that on hCG day [15]. Our results are with agreement to those that reported a positive correlation [3,4,10-21]. Endometrial thickness measured on the day of hCG administration was higher in cycles where pregnancy was achieved (mean 11.6 vs. 11.3 mm, respectively, p < 0.0001), but the difference is not of clinical significance, because results fell within the range of measurement error. When using a multiple logistic regression analysis to control all other confounding variables, we found an independent effect of endometrial thickness on PR. The uniqueness of this study is that it demonstrated a steady and gradual increase in PR as endometrial thickness increases. Many previous studies reported significant differences in PRs above and below a threshold thickness of 8 – 10 mm, but didn't show a continuous relationship such as we found [3,4,11,12,19]. Although we found a clear positive correlation between endometrial thickness and PR, our PR was 29.4% among patients with ≤ 6 mm endometrial thickness in contrast to Gonen et al 1990 who reported poor PR with endometrial thickness < 6 mm [14]. Furthermore, there were several reports of successful pregnancies resulting from cycles with endometrial thickness of ≤ 4 mm [22] indicating that a thin endometrium does not necessarily preclude the possibility of implantation. Hence cancellation of cycle based on a thin endometrium is unwarranted.

Some authors suggested a detrimental effect of endometrial thickness of ≥ 14 mm on PR [6]. Our results on the contrary, suggest that PRs are highest for patients with the thickest lining, and are consistent with other recent studies finding no reduction in PRs with very thick endometrium [16,23-25]. In fact there was a case report of a successful twin pregnancy after IVF with an endometrial thickness of 20 mm [26]. Limitations of our study, it is retrospective in nature, but all patients received hMG for stimulation, hence eliminating the bias that can result from different stimulation medications and their different effects on endometrial proliferation. Similarily the number of embryos transferred was limited to two, unless there was only one embryo available for transfer, to control its effect on PR. The poor predictive value of the ROC analysis makes it difficult to accurately determine a cut-off value, never the less, adequate endometrial development is required for pregnancy to occur, and PR were found to be higher when the endometrium reached at least 11 mm thickness.

## Conclusion

The results of the present study identified a positive linear correlation between endometrial thickness measured on hCG day and PR. Therefore, clinicians must pay close attention to endometrial development as well as to follicle growth. But again cancellation of embryo transfer based on a thin endometrial lining is unwarranted.

## Competing interests

The authors declare that they have no competing interests.

## Authors' contributions

All authors made substantial contribution to conception and design of the research. AA acquired the data and wrote the manuscript. KA performed the statistical analysis and interpreted the data, and SC critically revised the manuscript. Again all authors gave the final approval of the manuscript.
